# Coarse and Fine Culturable Fungal Air Concentrations in Urban and Rural Homes in Egypt

**DOI:** 10.3390/ijerph10030936

**Published:** 2013-03-06

**Authors:** Abdel Hameed A. Awad, Shawn G. Gibbs, Patrick M. Tarwater, Christopher F. Green

**Affiliations:** 1 Air Pollution Department, National Research Centre, P.O. Box 12622, Giza 11787, Egypt; E-Mail: abed196498@yahoo.com; 2 Department of Environmental and Health Research, the Custodian of the Two Holy Mosques Institute for Hajj and Umrah Research, Umm Al-Qura University, Makkah Al Mukarramah, 21421, Saudi Arabia; 3 College of Public Health, Department of Environmental, Agricultural and Occupational Health, University of Nebraska Medical Center, 984355 Nebraska Medical Center, Omaha, NE 68198, USA; 4 Paul L. Foster School of Medicine, Department of Biomedical Sciences, Texas Tech University Health Sciences Center, 5001 El Paso Drive, El Paso, TX 79905, USA; E-Mail: patrick.tarwater@ttuhsc.edu; 5 Science, Math and Engineering Division, University of Cincinnati Clermont College, Cincinnati, OH 45221, USA; E-Mail: greencf@ucmail.uc.edu

**Keywords:** air, fungi, homes, rural, urban

## Abstract

The main objective of the present study was to assess culturable airborne fungal concentrations, and types in different seasons. Two-stage viable impactor samplers were used with malt extract agar medium as the collection media. Culturable airborne fungal concentrations were collected indoors and outdoors of 43 homes in urban and rural environments from November 2008 to October 2009 in Egypt. Fungal concentrations were significantly higher in the rural environment than the urban environment. The median indoor and outdoor total fungal concentrations were 608 and 675 CFU/m^3^ in the urban environment and 1,932 and 1,872 CFU/m^3^ in the rural environment, respectively. The greatest concentrations were found in the autumn and spring season. Indoor and outdoor concentrations were significantly correlated (*P* < 0.001). The highest concentrations were observed in the fungal size range of <8 µm (fine fraction). The indoor/outdoor (I/O) ratios were not statistically different between seasons. *Alternaria*, *Aspergillus*, *Cladosporium*, *Penicillium* and yeasts were the predominant genera indoors and outdoors, and the abundance of genera varied by season and region. This study is of a potential interest as little reported research on the indoor fungal air quality from Egypt.

## 1. Introduction

Fungal organisms are common in indoor and outdoor air environments. They are known to induce irritation, infection, allergy, asthma, and toxic effects [1,2]. Fungi may produce mycotoxins that may cause several diseases in humans and animals. Knowledge of indoor airborne fungi is important, as various fungi could be common in indoor air of occupational and non-occupational buildings. 

In the recent years, assessment of buildings for evidence of indoor fungal growth has dramatically increased [3,4]. Many studies have focused on fungal concentrations in problematic or moldy contaminated buildings [5,6], and few references for normal buildings without mold damage [7,8]. Nevalainen *et al.* reported that the presence of indoor fungi and air quality problems could still occur in buildings without moisture or mold damage [9]. Fungal organisms are known to induce irritation, infection, allergy, asthma, and toxic effects [5,6].

The concentrations and types of airborne fungi depend on time of day, climatic conditions, geographical location and type of vegetation [10]. Airborne fungal concentrations in homes and schools ranged between 10^2^ and 10^4^ CFU/m^3^ in warm climates [11], and averaged 10^2^ CFU/m^3^ in cold subarctic climates [12]. Airborne fungal concentrations exceeded 10^4^ CFU/m^3^ in the moldy homes, and <200 CFU/m^3^ in the reference dwellings (without mold growth), with *Penicillium*, *Aspergillus*, *Cladosporium* and *Alternaria* being the common genera found [13]. In Poland, fungal concentrations averaged 60 CFU/m^3^ in healthy homes in winter, and >800 CFU/m^3^ in moldy homes in summer, with the maximum concentration reaching 17,000 CFU/m^3^ [14]. There is a growing interest in indoor air quality as the concentrations and prevalence of fungi in a particular region help identify the association between residential exposure, clinical diagnosis, and in the prevention of seasonal allergic diseases [15]. The aim of the present study was to assess concentrations, types and frequency of distributions of indoor and outdoor fungi in the air of urban and rural homes without a suspected or known problem, in order to gain data on home-indoor air quality in Egypt.

## 2. Experimental Section

### 2.1. Sampling Strategy and Sites

Air samples were collected indoors and outdoors, from 43 homes with no obviously visible fungal growth or existing water leaks (within the past six months). The samples were taken between 12.00 a.m. and 6.00 p.m. over a period of 12 months between November 2008 and October 2009.

Table 1 shows the housing characteristics of the investigated homes. Air samples were taken from 26 homes with an average size of 100 m^2^ in the urban environment across Greater Cairo (30°02" N and 31°20" E), and from 17 homes, with an average size of 130 m^2^, in the rural village located in "Dakahlia governorate" (30.5°–31.5° N and 30°–32° E), 150 km northeast of Cairo.

**Table 1:** Characteristics of the homes under the investigation.

	Characteristic	Urban (%)	Rural (%)
Age of building	1–5 years	7	0
	5–10 years	22	6
	10–15 years	15	6
	>15 years	56	88
Number of occupants	1–2 person	11	19
	3 person	4	0
	4 person	41	13
	≥5 person	44	69
Type of building	Single house	0	19
	Small complex (2–3 apartments)	0	81
	Big complex (>5 apartments)	100	0
Pets	Yes	4	50
	No	96	50

Temperature and relative humidity were measured during the course of the study. All homes had natural ventilation and no home had air-conditioning. Temperatures ranged between 20–42 °C indoors and 13–42 °C outdoors in the urban area, and 15–31 °C indoors and 12–33 °C outdoors in the rural area. Indoor and outdoor relative humidity ranged between 25–90% and 28–84%, respectively, in urban areas and 31–82% and 31–81% in the rural area. Egypt is a desert climate with very low rainfall (http://www.climate-zone.com/climate/egypt/). Egypt is characterized with moderate winter months, (December–February), warm spring (March–May) and autumn (September–November) months, and hot summer (June–August) months. Greater information on the environmental conditions can be found in the companion publication to this study that focused on bacterial bioaerosols [16].

### 2.2. Sampling of Fungi

Air samples were collected using the two-stage viable cascade impactor sampler (TE-10-160, Tisch Environmental, Cleves, OH, USA), separating particles into two size ranges: fine (<8 µm) and coarse (>8 µm) [2]. The particles ≥8 μm are usually deposited in the upper respiratory tract and can contribute to allergic rhinitis and asthma [15], however particles ≤8 μm can penetrate into the alveoli contributing to allergies [17]. 

Indoor samples were collected at a height of 1.5 m, the human breathing zone, above the floor level in the middle of the main common room or living room while outdoor comparison samples were taken approximately 5–10 m outside the entrance doors or on the roofs according to the location of homes inside the buildings. Sampling was limited so that no samples were collected within 48 h of a rain event or if daily wind speeds were predicted to exceed 15 km/h. The sampler was operated at the manufacturer recommended flow rate of 28.3 L/min for 2 min, to prevent overloading the collection plates. Petri dishes containing malt extract agar (BD, Sparks, MD, USA) supplemented with 50 ppm chloramphenicol (Oxoid, Hampshire, UK) were used to collect fungi. Each home was scheduled for sampling twice per season, and because of the short sampling time two consecutive indoor samples, at 12.00 and 15.00 p.m. where taken in parallel to the outdoor samples during every sampling event [2]. 

The Petri dishes were incubated at 28 °C for 5–7 days and colonies were counted with replicate plates used to calculate a mean. Positive-hole correction [18] was applied to the raw CFU recovered on each plate then used along with the sampling time and flow rate to calculate the concentration with final concentration expressed as colony forming units per cubic meter of air (CFU/m^3^). The recovery range for fungal organisms in this study ranged from a minimum of 17 to a maximum of 20,770 CFU/m^3^. Fungal isolates were purified and identified by direct observation on the basis of micro and macro-morphological features, reverse and surface coloration of colonies on Sabouraud dextrose agar, Czapek dox agar and Malt extract agar [2,19,20,21,22].

### 2.3. Statistical Analysis

The data were analyzed using descriptive statistics, such as median, mean, and percentiles. In addition, data analyses were conducted using non-parametric statistics, which do not require distributional assumptions (normal distribution). The Kruskal–Wallis test of the equality of medians is a non-parametric method was used to compare two or more populations (*P* < 0.05). The assumption for this test is that the samples from the different populations are independent random samples from continuous distributions with the distribution having a similar shape. Spearman’s rank (r_s_) correlation test was used to determine the linear relationships between the indoor and outdoor concentrations.

## 3. Results

### 3.1. Overall Concentrations

Table 2 shows the range, median, mean and median I/O ratios of fungal concentrations in the urban and rural homes. The concentrations ranged between 65 and 34,784 CFU/m^3^ in the urban area, and 67 and 16,492 CFU/m^3^ in the rural area. Fungal concentrations were to be higher in the rural environment than in the urban environment (*P* < 0.0002). Significant positive correlations (*P* ≤ 0.001) were found between the indoor and outdoor concentrations in both the urban (r_s_ = 0.79) and the rural (r_s_ = 0.81), and no significant differences were detected between both areas.

Fungal concentrations in the size fraction < 8 µm were higher than those of >8 µm, and constituted ~84% of the total organisms recovered. The median I/O ratios of fine and coarse fractions, respectively, were 0.789 and 0.91 in the urban area, and 1.14 and 1.07 in the rural area. I/O ratios for total fungal counts were 0.908 in the urban and 1.03 in the rural areas (Table 2). Generally, indoor fungal concentrations exceeded outdoor concentrations in the rural area.

**Table 2:** Indoor and outdoor range, mean and median fungal concentrations (colony forming units per cubic meter of air), and Indoor/Outdoor ratio for coarse, fine, and total organism sizes.

	Coarse (> 8µm)		Fine (< 8µm)		Total	
	Indoor	Outdoor	Indoor	Outdoor	Indoor	Outdoor
Urban (n = 26)						
Range	1–641	8–742	43–4,108	32–3,076	65–4,523	67–3,697
Mean	129	174	765	810	894	984
Median	101	128	495	541	608	675
I/O ratio ^a^	0.789		0.91		0.908	
Rural (n = 17)						
Range	40–2,744	1–4,604	67–32,040	120–14,264	111–34,784	360–16,492
Mean	572	613	3,273	2,854	3845	3467
Median	404	353	1,537	1,441	1,932	1,872
I/O ratio ^a^	1.14		1.07		1.03	

**^a^** the ratio was calculated by dividing the median of indoor concentration by the corresponding median of the outdoor concentration.

### 3.2. Seasonal Concentrations

The range, median, mean (CFU/m^3^) and I/O ratios of the total fungal concentrations in the different seasons are shown in Table 3. 

The greatest concentrations were found in the autumn (*P* < 0.05). The greatest I/O ratios were found in the summer in both the rural and urban environment. No significant differences (*P* > 0.05) were found between the I/O ratios between any season. The Spearman rank correlation analyses was utilized to evaluate correlations between environmental conditions (temperature and relative humidity) and fungal concentrations; however, no correlation was found.

**Figure 1:**
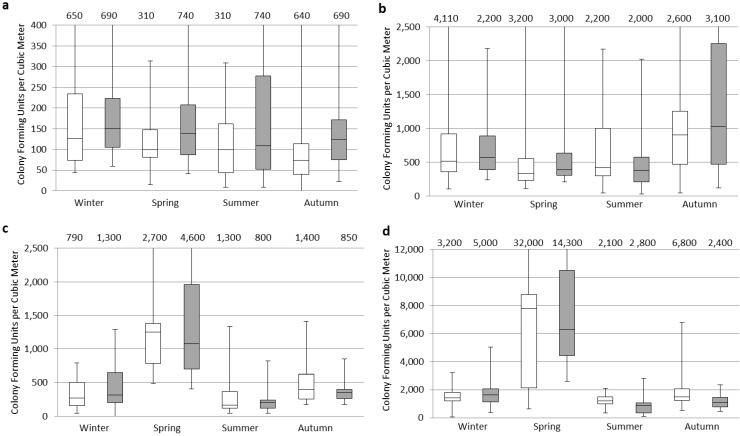
Culturable airborne fungal concentrations measured indoors and outdoors in the rural and urban sites in Egypt. The box plots represents indoors (white) and outdoors (grey) where: (**a**) Urban > 8 µm; (**b**) Urban < 8 µm; (**c**) Rural > 8 µm; and (**d**) Rural < 8 µm. Note: *Y*-axis is scaled differently in each graph.

The seasonal distributions of the fine and coarse fractions are shown in Figure 1. Fine fractions were generally greater than those of coarse fractions in all seasons in both urban and rural areas. The lowest concentration of the coarse fraction was found in the urban area. The highest median indoor and outdoor concentrations for the size fraction >8 µm were found in the winter (Figure 1(a)) and <8 µm in the autumn (Figure 1(b)), in the urban area, however the highest median concentrations for both size fractions were found in the spring (Figure 1(c,d)) in the rural area. 

**Table 3:** Total Indoor and outdoor range, mean and median fungal concentrations (colony forming units per cubic meter of air), and Indoor/Outdoor ratio by season.

	Winter		Spring		Summer		Autumn	
	Indoor	Outdoor	Indoor	Outdoor	Indoor	Outdoor	Indoor	Outdoor
**Urban (n = 26)**								
Range	239–4,523	317–2,868	212–2,376	283–3,697	72–2,482	67–2,600	65–3,285	194–3,236
Mean	988	887	751	829	761	706	1,066	1,500
Median	622	763	456	516	545	511	914	1,225
I/O ratio ^a^	0.81		0.88		1.06		0.746	
**Rural (n = 17)**								
Range	111–3,990	449–6,323	140–34,784	3,052–16,492	484–2,540	360–3,200	704–8,210	704–2,712
Median	1,707	2,028	8,644	7,636	1,480	988	1,827	1,509
Mean	25,649	20,730	22,402	21,073	13,355	9,782	19,458	17,143
I/O ratio ^a^	0.84		1.13		1.49		1.21	

**^a^** the ratio was calculated by dividing the median of indoor concentration by the corresponding median of the outdoor concentration.

### 3.3. Identification of Fungi

The counts and percentages of the predominant identified fungi are found in Table 4. The ranking of the most numerous genera were similar in both the urban and rural areas, with *Alternaria*, *Aspergillus*, *Cladosporium*, *Penicillium* and yeasts as the predominant types indoors and outdoors. 

**Table 4:** The total number of organisms recovered (CFU/m^3^) and percentages of the most prevalent fungal organisms recovered in urban and rural areas.

	Urban				Rural			
	Indoor		Outdoor		Indoor		Outdoor	
	CFU/m^3^	%	CFU/m^3^	%	CFU/m^3^	%	CFU/m^3^	%
*Alternaria*	1,279	1.4	2,099	2.2	5,849	2.4	8,370	2.0
*Aspergillus spp.*	30,387	33.9	26,819	27.8	18,967	7.9	13,579	3.2
*Cladosporium*	25,358	28.3	34,991	36.3	160,394	67.2	324,668	76.2
*Fusarium*	1,079	1.2	992	1.0	5,480	2.3	5,722	1.3
*Penicillium*	24,995	27.9	24,709	25.6	28,569	12.0	57,830	13.6
Yeasts	2,417	2.7	2,238	2.3	5,801	2.4	6,175	1.4
Other	3,993	4.5	4,600	4.8	13,707	5.7	9,696	2.3
Sum of all Samples	89,508	100	96,448	100	238,767	100	426,040	100

*Aspergillus flavus* and *Aspergillus niger* were the dominant *Aspergillus* species. *Aspergillus flavus* and *Penicillium* were higher in the urban homes, while *Alternaria* and *Cladosporium* in the rural homes. *Aspergillus spp.*, including *Aspergillus flavus* and *Aspergillus niger*, *Fusarium*, *Penicillium*, and yeasts were more numerous indoors, while *Alternaria* and *Cladosporium* were more numerous outdoors. Microsporium, Trichophyton, and water indicator fungi (*Stachybotrys* and *Ulocladium*) were found in low counts. *Aspergillus candidus*, *Aspergillus clavatus*, *Aspergillus lucknowensis*, *Aspergillus parasiticus*, *Drechslera* and *Trichoderma* as well as *Aspergillus egypticus*, *Gliocladium*, *Monilia*, *Stachybotrys* and *Trichophyton* were found indoors and not outdoors in the urban and rural homes, rerspectively.

Table 5 shows the counts of the predominant identified fungal types recovered by season. *Aspergillus spp.*, including *Aspergillus fumigatus* dominated in the summer, *Claodsporium* in the autumn and *Penicillium* in the winter. Yeasts were found in high counts in the summer. *Fusarium* was found in higher counts in the summer in the rural area. Although not in Table 5, *Drechslera* and *Epicoccum* were detected in the warm months, and *Ulocladium* and *Stachybotrys* in the wet months.

### 3.4. Frequency of the Occurrence of Fungal Genera by Season

The percentages of homes positive for identified fungal genera by season are shown in Table 6. *Alternaria*, *Aspergillus spp*, *Penicillium*, and *Cladosporium* were frequently found in all homes in every season. *Aspergillus flavus* was recovered indoor in percentages ranged from 73–96% in the urban, and 41–70% in the rural area, in different seasons, with the maximum in the spring and summer. *Aspergillus niger* was recovered in 100% and *Alternaria* in 15% of the homes investigated in the winter in the urban area. *Alternaria* was recovered in 82% of samples indoors and outdoors in the spring, *Cladosporium* in 100% in the winter, spring and autumn, and *Penicillium* in all homes in the autumn in the rural area. Although not shown in Table 5, *Stachybotrys* was found indoors in the winter (11.7%) in the rural area.

## 4. Discussion

Many studies have been performed to identify the concentrations and types of airborne fungal organisms in buildings all over the world. Almost all studies have focused on problematic buildings; however few reported research non-problematic building or on indoor air quality from Egypt.

In the present study, fungal concentrations were higher in the rural environment and may be a result of outdoor fungal sources, such as “animal shelters, solid-waste, composts, plant debris and vegetation”. The rate of air exchange may be higher in the rural homes where the rate of air ventilation from outdoor to indoor may help increase the indoor fungal concentrations. In milder climates the outdoor fungal organisms are the largest source of indoor concentrations of fungi and the outdoor concentration can routinely exceed of 10^3^ CFU/m^3^ [23]. In addition indoor fungi are directly dependent on building type, hygienic rules and ventilation [24]; life style and residential characteristics [15,25], and the rural homes in this study were characterized by higher ventilation rates, larger areas and dusty floors.

**Table 5:** The total counts (CFU/m^3^) for the indoor (In) and outdoor (Out) most prevalent fungal organisms recovered by season.

	Urban								Rural							
	Winter		Spring		Summer		Autumn		Winter		Spring		Summer		Autumn	
	In	Out	In	Out	In	Out	In	Out	In	Out	In	Out	In	Out	In	Out
*Alternaria*	120	135	333	959	737	975	89	30	351	819	4,424	6,380	1,040	880	34	291
*Aspergillus spp.*	7,726	6,804	3,230	3,860	11,062	7,989	8,334	8,136	4,889	3,896	1,488	800	6,997	5,104	5,214	3,779
*Cladosporium*	33	65	7,707	7,145	2,096	4,150	12,190	19,086	18,603	31,689	124,268	277,714	5,912	4,148	11,611	11,117
*Fusarium*	105	104	306	1,560	558	288	110	0	689	599	1,724	1,560	2,440	2,924	627	639
*Penicillium*	11,911	8,340	5,221	6,021	2,498	1,750	5,365	8,568	5,650	4,805	12,700	45,428	3,896	3,176	6,323	4,421
Yeasts	119	194	1,280	1,123	996	884	22	37	910	555	2,640	4,144	1,560	1,160	691	316
Other	1,170	1,154	8,354	8,181	2,921	5,318	13,594	20,394	23,174	35,863	125,957	278,914	8,678	5,358	16,523	14,161
Total	21,151	16,731	18,724	21,704	18,772	17,204	27,514	37,165	35,663	46,537	148,933	337,226	24,611	18,602	29,412	23,607

**Table 6:** Frequency of occurrence (n = 26-urban, n = 17-rural) as percent of homes positive for most prevalent fungal organisms for the indoor (In) and outdoor (Out) by season.

	Winter				Spring				Summer				Autumn			
	Urban		Rural		Urban		Rural		Urban		Rural		Urban		Rural	
	In	Out	In	Out	In	Out	In	Out	In	Out	In	Out	In	Out	In	Out
*Alternaria*	15	30	62.7	58.8	38	65	82	82	53.8	61.5	64	53	38	3.8	35	29
*Aspergillus spp.*	100	100	94.1	82.4	100	100	70.6	64.7	100	92.3	100	88.2	96.2	88.5	100	100
*Cladosporium*	84.6	88.4	100	100	65	53.8	100	100	34.6	42	64	53	84	88	100	100
*Fusarium*	19	15	47	53	30	53	93	76	30	38	88	88	11.5	0	58	64
*Penicillium*	96	100	94	100	92	84	94	94	46	53	100	88	50	88	100	100
Yeasts	15	15	70	47	84	69	82	100	46	50	64	64	1	2	47	41
Other	100	100	100	100	100	100	100	100	100	100	100	100	100	100	100	100

The indoor to outdoor ratio is used to compare the distribution and assess the potential sources of fungi. In the present study, most of the I/O ratios were close to one, with the slightly higher values in the rural area (Table 3) suggesting that not all indoor fungi originated from outdoors, which could be an indication of lesser indoor air quality. Hidden mold conditions, grains storage, dusty air and building materials may play a role in increased indoor fungal concentrations in the rural homes. The lowest I/O ratio that was found in the winter in the rural area may be attributed to less opening of windows that reduces outdoor infiltration of fungal organisms and decreases the presence of indoor dusty air from outdoor agricultural activities. On the other hand the lowest I/O ratio in urban area detected in autumn could be a result of increasing plant debris in the outdoor environment contributing to fungal concentrations outdoors. 

In the present study, fungal concentrations were found to be higher than those reported in previous studies around the world. Indoor and outdoor concentrations, respectively, averaged 82 CFU/m^3^ and 540 CFU/m^3^ across the US [26]; 89 CFU/m^3^ and 68 CFU/m^3^ in Cincinnati, OH, USA [27]; and 812 CFU/m^3^ and 1,042 CFU/m^3^ in Latrobe Valley, Australia [28]. Although results of several studies are similar to those of our study with indoor and outdoor concentrations, respectively, in 820 healthy (or reference homes) residential buildings ranged between 68–2,307 CFU/m^3^ and 400–80,000 CFU/m^3^ in the USA [29] and 264–17,788 CFU/m^3^ and 123–5,771 CFU/m^3^ in Mexico [30]. Moreover, in Egypt, fungal concentrations were found in the range of 10–10^2^ CFU/m^3^ in the indoor air of the Church of Saint Katherine Monastery in Sinai [31], and 52–124 CFU/m^3^ indoors and 25–222 CFU/m^3^ outdoors at the coastal buildings in Domitta [32]. 

There are no guidelines for airborne fungi, however a number of numeric guidelines have been proposed throughout the years [29,33,34,35,36], but none of them are currently widely accepted by the scientific community [7]. In comparison with the previously suggested guidelines, our findings indicated that the Egyptian^’^s homes had much higher than acceptable levels of fungi. The indoor air quality of the Egyptian^'^s homes may be considered a poor since fungal concentrations exceeded 500 CFU/m^3^ suggested by the World Health Organization [35] and Singapore [36] as well as 200 CFU/m^3^ by the American Conference of Governmental Industrial Hygienists [37]. In the present study, fungal concentrations in the size range of ≤8 µm (84.26%) was the predominant fraction. This was similar to the respirable fungal fraction concentrations that accounted for 70–80% of the total fungi in American and Taiwanese homes [2,38,39], and 79–98% in Mexico [30].

In most parts of the world the main core of fungal aerosols is likely to be similar, but the dominance of genera may differ from one area to another depending on geographical location, local sources, and climatic conditions [40]. Qualitative determination of fungi may be more useful than determining concentrations, as many species may have health effects. The frequent detection of *Aspergillus spp.*, *Penicillium*, and *Cladosporium* is attributed to their ready dissemination into the air. These findings in our study are similar to those observed in other geographical locations in Italy [41], Egypt [32,42], and Florida, USA [7]. 

Horner *et al.* [4] grouped fungi into three categories with different ecological relevance: (1) phyloplane fungi (*Cladosporium*, *Curvularia* and *Alternaria*); (2) soil fungi (*Penicillium*, *Paecilomyces* and *Aspergillus*), and (3), water indicator fungi (*Chaetomium*, *Stachybotrys* and *Ulocladium*). In the present study water indicator fungi were found in low counts, and their presence associated with rain time or the presence of damped materials, however phyloplane and soil fungi were found in higher counts in the rural environment.

*Cladosporium* contains species that commonly grow indoors (*C. sphaerospermum*) and outdoors (*C. cladosporioides* and *C. herbarum*) [43]. Yeasts and *Cladosporium* dominate healthy homes while *Absidia* and *Alternaria* are more likely in moldy homes, and *Penicillium* and *Cladosporium* in both healthy and moldy homes [14]. *Alternaria* has an affinity for outdoor substrates, and when its I/O ratio exceeds one, this could indicate the presence of abnormal indoor conditions. The presence of *Aureobasidium* and *Eurotium* in rural homes is an indication of the presence of cellulotic materials and bad storage conditions, respectively. Moreover, the presence of some fungal species in indoor not outdoors mainly *Stachybotrys* may indicate hidden mold conditions particularly in the rural homes.

Regarding season, *Cladosporium*, *Penicillium* and yeasts dominated in the winter, while *Aspergillus spp.* in the summer. High concentrations of *Aspergillus spp.* and *Alternaria* in the autumn are associated with the decaying vegetable materials [44]. *Penicillium* was found in the highest concentration in the months with low temperatures, as precipitation seemed to optimize their sporulation [45]. Hargreaves *et al.* [46] isolated maximum concentrations of *Alternaria* in the spring, as the suitability of humidity and temperature and vegetation debris [25]. 

Cladosporium, Penicillium and Alternaria could increase the risk of asthma and allergic rhinitis and allergic alveolitis [47]. *Acremonium*, *Alternaria*, *Aspergillus*, *Cladosporium*, *Fusarium*, *Paecilomyces*, *Penicillium*, *Stachybotrys* and *Trichoderma* are well known mycotoxin producers [48]. *Aspergillus flavus* and *Aspergillus fumigatus* can lead to aspergillosis [49], and *Microsporium* and *Trichophyton* are agents of dermatophytoses [50]. As the potential implications of the fungal contamination on health have not been studied in Egypt, it should to be reported that exposure to such concentrations and types is a risk factor for resident’s respiratory symptoms.

In the present study, the results represent the normal mycoflora in non-problem homes and describe the typical background concentrations in the indoor air environment. It should be mentioned that, the main limitations in the present study were: (1) short indoor sampling times that may not provide representative samples or accurately reflect exposures; (2) outdoor samples may not be representative since the apartment complexes restricted the airflow; and (3) geographical regions were large and could contain localized sources, and (4) explanations in discussion section regarding fungal concentration variations are speculation and not supported by local measurements. However, we believe that all buildings should be thoroughly inspected for visible signs of moisture. In addition good ventilation, household maintenance, minimizing dust generation and applying a proper and routine cleaning should be applied.

## 5. Conclusions

Indoor culturable airborne fungi were found in concentrations relatively similar to those outdoors of the homes. Fungal concentration and composition varied by season and no significant differences were found between the rural and urban homes and indoors and outdoors concentrations. Fungal organisms of the size fraction <8 µm represented 84.26% of the total fungal counts with the greatest counts found in the spring and autumn. I/O ratios slightly exceeded one for coarse, fine and total fungi in the rural area, and the highest ratios were found in the summer season for both rural and urban environments. Knowledge on airborne fungi is important as it is considered a potential public health problem and data can be used as a base to develop criteria for assessing indoor air quality in Egypt. Studies regarding fungal concentrations and types in buildings with moisture and damp materials are needed in the future.
